# Microarray analysis of the *Escherichia coli* response to CdTe-GSH Quantum Dots: understanding the bacterial toxicity of semiconductor nanoparticles

**DOI:** 10.1186/1471-2164-15-1099

**Published:** 2014-12-12

**Authors:** Juan P Monrás, Bernardo Collao, Roberto C Molina-Quiroz, Gonzalo A Pradenas, Luis A Saona, Vicente Durán-Toro, Nicolás Órdenes-Aenishanslins, Felipe A Venegas, David E Loyola, Denisse Bravo, Paulina F Calderón, Iván L Calderón, Claudio C Vásquez, Thomas G Chasteen, Desiré A Lopez, José M Pérez-Donoso

**Affiliations:** Bionanotechnology and Microbiology Lab, Center for Bioinformatics and Integrative Biology (CBIB), Universidad Andres Bello, Santiago, Chile; Facultad de Ciencias Químicas y Farmacéuticas, Universidad de Chile, Santiago, Chile; Facultad de Química y Biología, Universidad de Santiago de Chile, Santiago, Chile; Facultad de Odontología, Universidad de Chile, Santiago, Chile; Laboratorio de Microbiología Molecular, Facultad de Ciencias Biológicas, Universidad Andres Bello, Santiago, Chile; Fraunhofer Chile Research, M. Sánchez Fontecilla 310 piso 14, Santiago, Chile; Department of Chemistry and Texas Research Institute for Environmental Studies, Sam Houston State University, Huntsville, Texas 77340 USA; Centro Interdisciplinario de Neurociencia de Valparaíso, Facultad de Ciencias, Universidad de Valparaíso, Valparaíso, Chile

**Keywords:** Toxicity mechanism, Transcriptomic response, Oxidative stress, Cadmium, Nanoparticles

## Abstract

**Background:**

Most semiconductor nanoparticles used in biomedical applications are made of heavy metals and involve synthetic methods that require organic solvents and high temperatures. This issue makes the development of water-soluble nanoparticles with lower toxicity a major topic of interest. In a previous work our group described a biomimetic method for the aqueous synthesis of CdTe-GSH Quantum Dots (QDs) using biomolecules present in cells as reducing and stabilizing agents. This protocol produces nanoparticles with good fluorescent properties and less toxicity than those synthesized by regular chemical methods. Nevertheless, biomimetic CdTe-GSH nanoparticles still display some toxicity, so it is important to know in detail the effects of these semiconductor nanoparticles on cells, their levels of toxicity and the strategies that cells develop to overcome it.

**Results:**

In this work, the response of *E. coli* exposed to different sized-CdTe-GSH QDs synthesized by a biomimetic protocol was evaluated through transcriptomic, biochemical, microbiological and genetic approaches. It was determined that: i) red QDs (5 nm) display higher toxicity than green (3 nm), ii) QDs mainly induce expression of genes involved with Cd^+2^ stress (*zntA* and *znuA*) and tellurium does not contribute significantly to QDs-mediated toxicity since cells incorporate low levels of Te, iii) red QDs also induce genes related to oxidative stress response and membrane proteins, iv) Cd^2+^ release is higher in red QDs, and v) QDs render the cells more sensitive to polymyxin B.

**Conclusion:**

Based on the results obtained in this work, a general model of CdTe-GSH QDs toxicity in *E. coli* is proposed. Results indicate that bacterial toxicity of QDs is mainly associated with cadmium release, oxidative stress and loss of membrane integrity. The higher toxicity of red QDs is most probably due to higher cadmium content and release from the nanoparticle as compared to green QDs. Moreover, QDs-treated cells become more sensitive to polymyxin B making these biomimetic QDs candidates for adjuvant therapies against bacterial infections.

**Electronic supplementary material:**

The online version of this article (doi:10.1186/1471-2164-15-1099) contains supplementary material, which is available to authorized users.

## Background

Quantum dots (QDs) are fluorescent semiconductor nanoparticles composed of a metallic core and a surface layer of organic and/or inorganic molecules [1, 2]. The metallic core determines the novel properties of QDs (spectroscopic, catalytic, etc.) and the surface layer modulates their toxicity and solvent stability [3, 4]. One of the most intrinsic properties of QDs is their size-dependent emission, a mechanical quantum effect controlled by the nanoparticle size. As the nanocrystal grows, its fluorescence emission peak can change from shorter wavelengths in small nanoparticles (blue or green emission), to longer wavelengths for bigger nanoparticles (yellow or red). These unique properties allow them to be used in nanoelectronics and biomedical research [5, 6]. Cadmium telluride QDs have several properties such as broad light absorption, narrow emission and photostability, which make them an interesting material for medical treatments in photodynamic therapy when conjugated with photosensitizers and targeting probes, molecular imaging and therapeutic targeting, among other applications in nanomedicine [7–9].

Most QDs described so far exhibit some toxicity; however, many studies have proven that using thiols as stabilizing ligands decrease their toxic effects [10–12]. Thiols also render QDs water-soluble thus favoring their conjugation with antibodies, nucleic acids and proteins, increasing their applications [13, 14]. Based on their bacterial toxicity, CdTe and other nanoparticles (iron, silver and gold) have been tested as antibacterial agents, alone or conjugated with antibiotics [15–18].

Given the wide range of applications that QDs display, it is of major importance to determine the effects that these nanoparticles have in eukaryotic and prokaryotic organisms. To date, several studies regarding QDs toxicity in different cell lines have been published [19–21], but just a few of them have focused on bacterial toxicity. In these reports, cadmium QDs display different degrees of toxicity, causing a variety of cellular damages at concentrations ranging from 1 nM to 3 μM (Table 1). The available evidence reported to date regarding cadmium-QDs toxicity reveals that the effects on bacterial cells are mostly related to membrane damage and reactive oxygen species (ROS) generation, and just a few reports have suggested the importance of Cd^2+^ ions (Table 1). Furthermore, only a few studies have compared cytotoxicity of QDs with the same core but different size, indicating that smaller QDs display higher toxicity than larger nanoparticles. It has been reported that the size of CdTe QDs, contributes to the cellular toxicity of nanoparticles, with smaller QDs exhibiting more toxicity than larger nanoparticles [22]. The same effect was seen in different sized CdTe and CdSe QDs, where smaller nanoparticles exerted the highest toxicity in *E. coli* cells and other cell lines [23, 24]. On the other hand, in 2011 Yang et al. analyzed the transcriptional response of *Pseudomonas stutzeri* exposed to chemically-synthesized QDs and observed changes in the transcription profile of 7 genes including some denitrification genes (*narG*, *napB*, *nirH* and *norB*) and the up-regulation of the superoxide dismutase gene (*sodB*), suggesting the production of ROS [25]. Also, analyses, made by the same group on *P. aeruginosa* PAO1 exposed to CdSe QDs, determined the expression of a few selected genes related to heavy metals and oxidative stress response [20]. Despite all these antecedents, no global transcriptional analysis of bacteria exposed to QDs has been reported to date.

**Table 1:** **Overview of Cd-QDs toxicity on bacteria**

QD	Capping layer	Microorganism	Reported cellular effect	Concentration tested	Reference
CdSe	Carboxyl coated	*E. coli, Bacillus subtilis, P. aeruginosa*	Growth inhibition	80 nM	[26]
CdTe	Cystine	*E. coli*	Growth inhibition and ROS generation	10- 40 nM	[27]
CdTe	3-mercaptopropionic acid	*E. coli, P. aeruginosa, Bacillus subtilis, Staphylococcus aureus*	Membrane damage and ROS generation	200- 300 nM	[28]
CdTe	Thioglycolic acid, glutathione	*Cupriavidus metallidurans, E. coli, Shewanella oneidensis, B. subtilis*	Growth inhibition and bacterial filamentation	1-100 nM	[29]
CdSe and CdTe	Cysteine and mercaptoacetic acid	*Photobacterium phosphoreum*	Affects luminescence metabolism	0,1-800 μg/mL	[30]
CdTe	Cysteine	*E. coli, P. aeruginosa, B. subtilis, S. aureus*	Affects electron transfer	40-100 nM	[31]
CdTe	Mercaptoacetic acid	*E. coli*	Membrane damage	50- 1 000 nM	[32]
CdTe	Thioglycolic acid	*E. coli*	Reduces viability and ROS generation	120 μg/mL	[33]
CdTe	3-mercaptopropionic acid	*E. coli*	ROS generation	1 mM	[15]
CdSe/CdZnS	Carboxyl, Polyanionicpolymaleic anhydride-alt-1-octadecene and polycationicpolyethylenimine	*Pseudomonas stutzeri*	Affects denitrification process and ROS generation	10-500 nM	[34]
CdSe	Octadecylamine	*E. coli*	No toxicity	0.01-100 μg/mL	[35]
CdSe/ZnS	Carboxyl coated and uncoated	*P. aeruginosa*	Oxidative stress and expression of cadmium efflux systems	20-60 nM	[20]
CdTe	3-mercaptopropionic acid	*E. coli*	Growth inhibition, membrane damage and cadmium release from QDs	20-120 nM	[23]
CdTe	3-mercaptopropionic acid, glutathione, N-acetyl cysteine	*E. coli*	Growth inhibition and membrane damage	100-3 000 nM	[36]
CdSe/CdS	Mercaptosuccinic acid	*E. coli*	Oxidative stress and cadmium release from QDs	5-2 000 nM*	[37]

*QDs concentrations were determined by using the molar extinction coefficient reported for CdTe or CdSe.

Recently, our group developed a biomimetic method to synthesize GSH-coated CdTe QDs (CdTe-GSH). QDs synthesized by this method display high biocompatibility and stable fluorescence varying from green to red emission as the size of the NPs grows (3 to 5 nm, respectively) [38–40]. Even though these QDs present low toxicity, they generate some degree of necrosis in cell lines [39] and inhibit bacterial growth [38]. Conversely to most nanoparticles reported to date [22–24], small size green biomimetic QDs display lower toxicity than red QDs as a consequence of a lower cadmium content and higher amount of GSH in the external layer [38, 39].

In this work, the *E. coli* global transcriptional response to green and red CdTe-GSH QDs was determined. Genetic, biochemical and microbiological experimental approaches were used to validate microarray results and to shed light on QDs toxicity in *E. coli*. Based on these results a toxicity mechanism was proposed and the use of QDs as antibiotic adjuvants was evaluated.

## Results and discussion

### Microarray analysis of QDs-treated cells

To evaluate the toxicity of green and red CdTe-GSH QDs in *E. coli*, MICs (minimal inhibitory concentrations) in the presence of these QDs were determined. *E. coli* MICs of green and red QDs are 2 000 and 125 μg/mL, respectively, confirming that these CdTe-GSH QDs display differential toxicity against *E. coli*, with red QDs clearly more toxic than green nanoparticles.

To understand the bacterial global response to QDs of different size, gene expression changes in *E. coli* were determined by microarray analysis after 15 min exposure to 50 μg/mL red or green QDs. This concentration was selected based on previous results indicating that growth of *E. coli* cultures amended with 50 μg/mL red or green biomimetic QDs in exponential phase was not affected [38].

**Figure 1:**
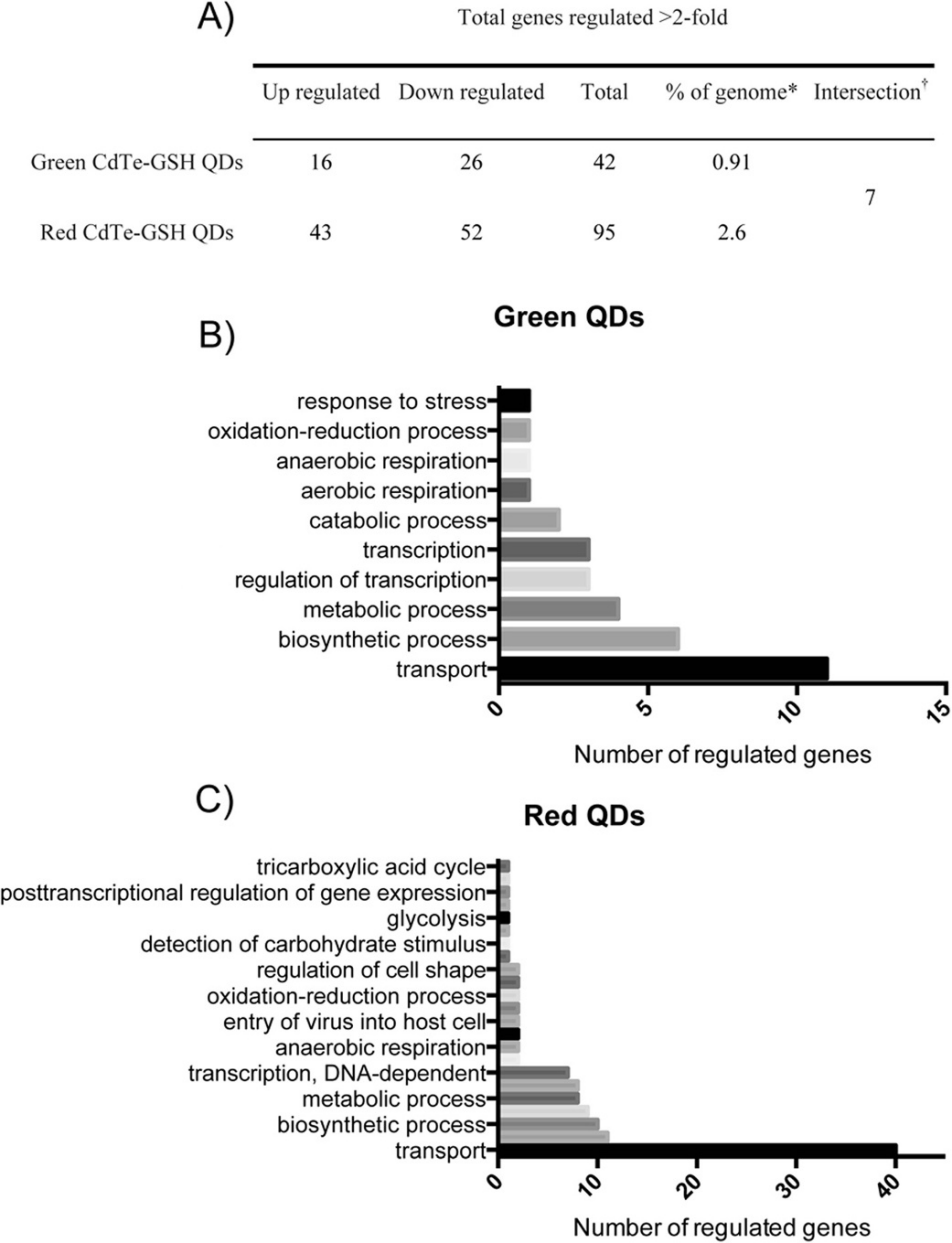
**Gene regulation in E. coli exposed to green or red QDs. (A)** Summary of total genes regulated in response to green, red and both QDs. Bar charts showing the number of regulated genes sharing specific Gene Ontology (GO) terms after red **(B)** and green QDs **(C)** treatment.*Whole genome reference set containing 4 619 E. coli genes. †Genes found in both treatments.

An *E. coli* transcriptomic analysis of 4 619 open reading frames —after QDs exposure— indicated the induction or repression of several genes (Additional file 1: Tables S3 and Additional file 2: Table S4). Microarray data were validated by comparing the expression ratio of 14 genes (*adhE*, *clpB*, *dnaK*, *hfq*, *kpdE*, *marR*, *minD*, *nfrB*, *ompW*, *soxS*, *trxC*, *wzxE*, *zntA* and *znuA*) with the results of expression determined by real-time PCR (Additional file 3: Figure S1). Microarray results showed that 95 and 42 genes are regulated in response to red and green QDs, respectively (Figure 1A). Thus, 2.6% of the genome is modulated by red QDs while only 0.9% is regulated under green QDs treatment (Figure 1A). Furthermore, 7 genes were regulated by both treatments (Figure 1A, Additional file 4: Table S5). Gene Ontology (GO) analysis indicated that the most affected processes in exposure to green or red QDs are related to transport, biosynthesis and metabolism (Figure 1B and C). However, in the case of red QDs treatment, a high modulation in genes related to transport (almost 4-fold higher than that observed with green QDs) and a moderate effect on genes involved in glycolysis and tricarboxylic acid cycle were observed (Figure 1C).

Several QDs-modulated genes determined in this transcriptomic analysis were previously reported in Cd^2+^-exposed *E. coli* through microarray experiments (*trxC*, *soxS*, *zntA*, *adhE*, *dnaK*, *hfq*, *clpB*, *marR*, *sucA*, *cspA* and *cspB*), confirming the relevance of Cd^2+^ release in biomimetic QDs toxicity [41]. Moreover, a subset of genes modulated by these QDs and not previously associated with Cd^2+^ transcriptional response was determined (*ybgK, clpS*, *hylC*, *yfcF*, *nrfC*, *ftn*, *feoB*, *nikAD*, *ompF, ompW*, among others). Transcriptional modulation of these genes may reflect the existence of a different and still unreported Cd-independent toxicity mechanism of CdTe-GSH QDs.

Red QDs induce the transcription of a large set of genes related to oxidative stress. Also, an increased level of transcripts related with protein re-assembly, degradation and disaggregation [41–44] was determined in cells exposed to QDs: *clpS*, *cplB* and *dnaK* induced by red and *hycl* by green QDs. Protein degradation could be a result of oxidative damage caused by treatment with QDs. This idea is supported by the increased transcription of *trxC*, encoding the oxidative defense-related thioredoxin 2 (Trx2) [45], in cells exposed to red QDs. Trx2 is involved in the thiol redox homeostasis and its augmented expression has been related to Cd^2+^ exposure and oxidative stress [46, 47]. The induction of SoxS*,* a transcriptional factor activated by the oxidative stress response regulator SoxR, was also observed in cells exposed to biomimetic QDs. This factor regulates the transcription of several genes involved in the defense against oxidative stress, such as *sodA* (superoxide dismutase), and modulates other cellular processes, like membrane permeability, by regulating the expression of efflux systems (e.g. AcrA-TolC) and outer membrane proteins (e.g. *ompF*) [48].

Other evidences of oxidative damage—observed exclusively in red QDs—comes from the increased expression of genes related (directly or indirectly) to oxidative stress response (Additional file 1: Tables S3 and Additional file 2: Table S4). Among them are: *adhE*, encoding for alcohol dehydrogenase E which has been associated with resistance to protein oxidation [49]; *hfq*, which is involved in post-transcriptional regulation of oxidative and envelope stress response [50, 51]; and *marR*, a transcriptional regulator of genes involved in the defense against oxidative stress and several other stresses [52]. All these genes have been previously determined as part of the cadmium regulon response [41]. Newly described genes responding to QDs exposure such as *yfcF*, whose product has glutathione peroxidase activity [53], and *lysU*, encoding an oxidative stress related alarmone [54], could be part of a novel CdTe-GSH QDs response.

A source of ROS generation is free Fe^2+^ that can produce hydroxyl radical through the Fenton reaction inside the cell [55]. To avoid this Fe^2+^-dependent Fenton reaction after red QDs exposure, *E. coli* decreases *paaD* and *nrfC* expression, leading to reduced intracellular levels of iron-sulfur clusters [56, 57]; it also induces *ftn* transcription, which would reduce available iron in the cytoplasm [58]. In exposure to green QDs, there is a repression of the *feoB* iron transporter, thus shutting down the entrance of iron into the cell cytoplasm. All these results may reflect *E. coli*’s strategies to defend itself against CdTe-GSH QD-generated oxidative damage, a result that is in agreement with previous reports on Cd-based NPs [15, 23, 27, 28, 32–34, 36, 37].

Another effect observed after E. coli exposure to red QDs was related to sugar and amino acid metabolism. The expression of genes related to several metabolic pathways seems to be modulated, favoring the accumulation of antioxidant metabolites such as pyruvate (e.g. sdaA, favoring the serine deamination to pyruvate and ammonia; and *alaC*, deaminating alanine to glutamate and pyruvate). Increased pyruvate content may be related to a metabolic reconfiguration since this metabolite is a known ROS scavenging agent [59]. Moreover, lower sucA expression may favor the accumulation of 2-oxoglutarate, which is also associated with oxidative stress protection [60]. These results suggest a concerted metabolic response to increase protection against oxidative damage.

QDs stress responses associated with metal transporters were observed in *E. coli* exposed to both green and red nanoparticles. For instance, higher *zntA* (a metal efflux pump) [61] and *znuA* (an influx pump of zinc) [62] transcript levels were observed upon QDs exposure. ZnuA, a periplasmic zinc-binding protein that allows the influx of Zn^2+^, has been described as a membrane and macromolecules stabilizer [63, 64], and also as antioxidant [65]. On the other hand, down regulation of *nikAD* in response to CdTe-GSH QDs could be explained by the possibility of this nickel transporter allowing the influx of Cd^2+^ and/or other pro-oxidant metals.

When QDs come into close contact with the bacterial cell, the first interaction should be with the cell envelope, a multilayered complex structure that serves as the first line of defense against many environmental stresses. It has been reported that GSH-coated QDs have a greater effect on membrane function than other thiol-coated QDs, probably due to GSH lipophilicity [36]. In this context, nanoparticle damage to the bacterial membrane and Cd-induced stress could act in conjunction to affect membrane function, thus explaining why this envelope stress response has not been determined by other Cd microarray studies [41, 66]. Some secondary transporters are suppressed suggesting membrane damage or a mechanism preventing metal entrance. Among them, *lamb* and *lldP* encoding a lactate permease; *malF, malM and malE* coding maltose uptake systems; and *hisQ,* involved in histidine, lysine and arginine uptake. Also, a number of transporters involved in the entry of oxidant species such as *ompF* and *ompW*, among others, are down-regulated in QDs-exposed cells [67]. The down-regulation of the major porin OmpF during red QDs treatment reveals that there is a major shut down of the entry of several molecules to the cell. This kind of effect has been reported to be a posttranscriptional repression, mediated by *micF*, an antisense RNA regulated positively by activation of the SoxRS regulon, in response to redox stress [48]. On the other hand, green QDs down-regulate the expression of OmpW*,* an outer membrane protein that allows the incorporation of oxidants such as H_2_O_2_ and NaOCl; accordingly it is well known that OmpW is down-regulated when the cell faces oxidative stress [67].

**Figure 2:**
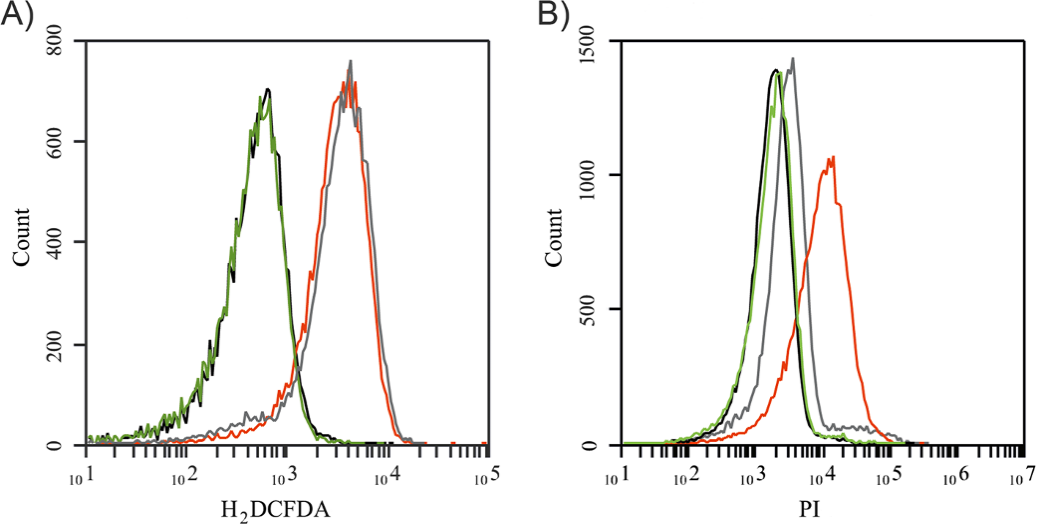
**QDs-mediated ROS production and membrane damage in** ***E. coli*** **. (A)** H2DCFDA-FACS histogram for ROS detection and **(B)** PI-FACS histogram for membrane damage detection. Cells were treated with green (green line) or red (red line) QDs during 30 min. Black and grey lines represent non-treated cells and cells exposed to 50 mM H2O2 (positive control), respectively. Results shown are representative of three independent experiments.

In general, microarray results indicated that QDs exposure modulates the expression of genes involved in membrane and oxidative stress defense, metal transport and metabolic processes (Figure 2A and B, Additional file 1: Tables S3 and Additional file 2: Table S4).

### ROS generation and membrane damage

**Figure 3:**
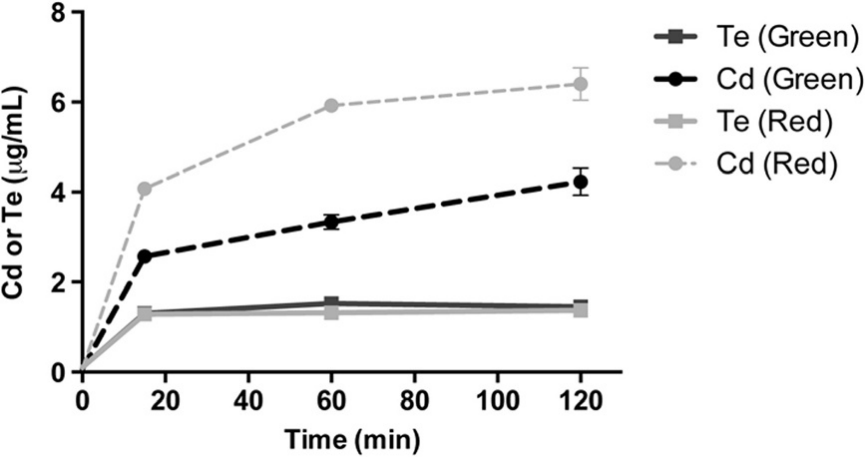
**Release of cadmium by green and red QDs.** *In vivo* cadmium uptake kinetics of cells exposed to QDs. Values are mean ± SD. Experiments were repeated three times and asterisks represent statistical differences between treatments (***p < 0.001).

Based on microarray results we decided to evaluate the generation of ROS and membrane damage in *E. coli* cells exposed to biomimetic QDs. The fluorescent probes H_2_DCFDA and PI were used in flow cytometry experiments to detect ROS and membrane damage, respectively. Only red QDs increased ROS production and membrane damage at 50 μg/mL, while green QDs do not produce any effect at the same concentration (Figure 3) or even at 500 μg/mL (data not shown). Obtained results are in agreement with the transcriptional response determined for red and green biomimetic QDs, and confirm that these QDs produce differential effect in cells mostly related to oxidative damage.

### *In vivo*and *in vitro*release of metal

Based on microarray results, and to evaluate if QDs toxicity is related to metal release, ICP-AES analysis of intracellular Te and Cd was carried out in cells previously exposed to 50 μg/mL QDs. After 2 h treatment, cells exposed to green or red QDs accumulated similar amounts of Te (1.46 ± 0.02 and 1.38 ± 0.03 μg/mL, respectively) (Figure 3). In addition, similar uptake kinetics were observed in cells exposed to both kinds of QDs, reaching maximal incorporation after 15 min exposure (Figure 3). These results suggest that Te is not related to the differential effect observed between exposure to green and red QDs and supports the idea that Te is not the main element responsible of CdTe-GSH QDs toxicity in E. coli. Cd accumulation increased over time in cells exposed to green or red QDs; however, a higher uptake was observed in cells exposed to red QDs (Figure 3). After 2 h, Cd accumulation reached 6.4 ± 0.29 and 4.23 ± 0.25 μg/mL for red and green QDs, respectively (Figure 3). Intracellular Cd content was always higher in cells exposed to red QDs, a result that is in agreement with the increased Cd content previously determined in this nanoparticle [39].

Next, experiments were carried out to evaluate if Cd release is an intrinsic property of Cd-containing QDs or is a consequence of their interaction with bacterial cells. Cd released by green and red QDs in the absence of cells was determined by FAAS (flame atomic absorption spectrometry). Biomimetic QDs released almost undetectable amounts of metal at 50 μg/mL exposure, so QDs concentrations of 1 000 μg/mL were tested. Results indicated that both QDs sizes released Cd in the absence of bacteria; however, red QDs released a statistically significant 2.5-fold more metal than green QDs (0.17 ± 0.01 versus 0.49 ± 0.04 μg/mL Cd^2+^, respectively). This result demonstrates that CdTe-GSH QDs release small amounts of Cd passively into the medium independently of the presence of bacterial cells, with red nanoparticles releasing higher amounts of this toxic element. Interestingly, results suggest that nanoparticle dismantling is favored in the presence of bacterial cells, since Cd was detected inside bacteria exposed to 50 μg/mL QDs (a concentration in which Cd release is undetectable *in vitro*).

In previous work, XPS (X-ray photoelectron spectroscopy) experiments indicated that cadmium species on the surface of green and red biomimetic QDs are CdO2 and CdO, respectively [40]. In addition, since red QDs release higher levels of cadmium (Figure 3) and display low levels of GSH, it is unlikely that Cd-GSH complexes could dissociate from the NP as has been reported for CdSe-GSH QDs [37]. In this context, QDs toxicity most probably depends on the release of Cd2+ or cadmium oxides, as has been reported for other CdTe QDs [29]. Taken together, the results described here could explain part of the CdTe-GSH toxicity and the differential effect observed between red and green QDs.

### QDs toxicity for mutant *E. coli*strains

**Figure 4:**
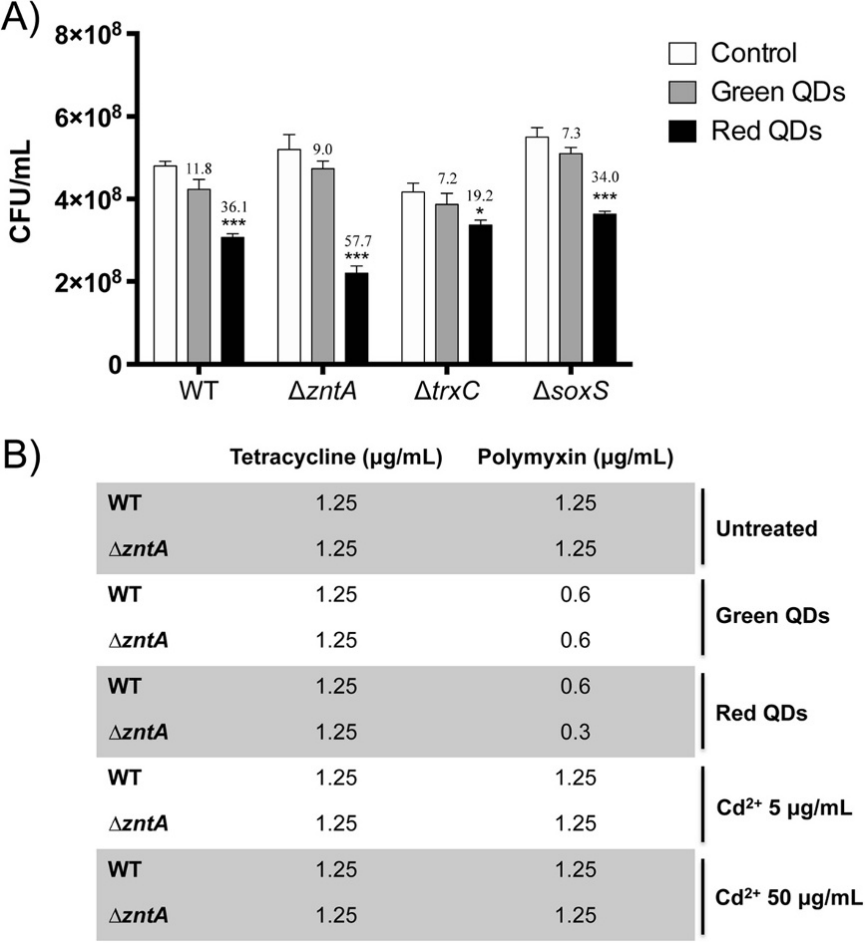
**Effect of QDs on viability and antibiotic susceptibility of** ***E. coli*** **wt and mutant strains. (A)** CFUs of *E. coli* wt, ∆*zntA,* ∆*trxC* and Δ*soxS* strains after 30 min exposure to green or red QDs. Numbers indicate the viability decrease percentage between control and QDs-treated cells. Values are mean ± SD. Experiments were repeated three times and asterisks represent statistical differences between control and treatments (*p < 0.05, ***p < 0.001). **(B)** MICs to tetracycline and polymyxin B for *E. coli* wt and ∆*zntA* strains pre-treated 15 min with cadmium, green or red QDs (for details see Methods).

Microarray analysis and metal release experiments suggests that CdTe-GSH QDs toxicity is mainly a consequence of Cd release and oxidative stress. To confirm this hypothesis, the viability of E. coli wild type and mutant strains on genes involved in Cd response (∆*zntA*) or oxidative stress defenses (∆*trxC* and ∆*soxS*) was assessed after exposure to QDs. All tested genes were positively regulated under QDs stress in the microarray study (Additional file 1: Tables S3 and Additional file 2: Table S4). At exposure levels of 50 μg/mL green QDs, cell viability was insignificantly affected in all strains (Figure 4A); however, viability was significantly affected with red QDs at this concentration. This effect was stronger in ∆*zntA* cells, which lack a gene involved in Cd export (57.7% decrease in viability as compared to untreated cells, Figure 4A) [41]. Surprisingly, susceptibility of ∆*soxS* and ∆*trxC* strains to QDs was similar to that exhibited by the wild type strain, suggesting that cells probably have other QDs response systems that can deal with oxidative damage when either of those genes is not present. These results indicate that QDs mainly affect the viability of cells lacking Cd response systems, confirming that Cd2+ stress is important in CdTe-GSH QDs toxicity.

### Effect of antibiotics on QDs-treated cells

Given that the microarray results indicated that one of the sources of CdTe-GSH QDs toxicity in *E. coli* is oxidative stress and membrane damage, the antibiotic susceptibility in bacteria previously exposed to green or red QDs was evaluated. Polymyxin B, a ROS generating antibiotic affecting cell membrane, and tetracycline, a bacteriostatic inhibitor of protein synthesis were used. A MIC of 1.25 μg/mL was determined for tetracycline and polymyxin B for untreated *E. coli* wt and ∆*zntA* strains (Figure 4B). No effect of QDs pre-treatment for the tetracycline MIC was observed. However, when cells were pre-treated with green or red QDs, polymyxin B MIC decreased to 0.6 μg/mL in the wild type strain. A higher effect on polymyxin B antimicrobial activity was observed with red QDs only for the ∆*zntA* strain, an outcome that is in agreement with the higher toxicity of red QDs determined in this work. These results confirm that pre-treatment of bacterial cells with QDs render them more susceptible to polymyxin B and that a mutant—lacking defenses against cadmium injuries—became even more sensitive to this antibiotic. Based on this, the effect of Cd^2+^ pre-treatment on *E. coli* polymyxin B MIC was evaluated. Surprisingly, no enhancement of polymyxin B antimicrobial effect was observed after Cd^2+^ pre-treatment (5 or 50 μg/mL) on wt and ∆*zntA* strains (Figure 4B). This result strongly suggests that increased polymyxin B toxicity observed after QDs pre-treatment is not a direct consequence of Cd release.

Recently, Yang et al. [20] reported that the resistance of *P. aeruginosa* PAO1 to antibiotics increased when the cells were pre-treated with CdSe/ZnS QDs, due to the activation of response mechanisms before the antibiotic was added. No such effect was observed in biomimetic CdTe-GSH QDs, probably as a consequence of their different composition and the synthetic procedures used for QDs production. In agreement with our results, other studies concluded that the effectiveness of antibiotics like penicillin G, amoxicillin and erythromycin, increase in the presence of metal nanoparticles [16–18]. Our results indicate that the increased polymyxin B toxicity observed in QDs-treated cells is not associated with cadmium release. These results are in agreement with the microarray analysis indicating that QDs toxicity involves other mechanisms of damage, such as envelope and oxidative stress, among others. Based on the low toxicity to eukaryotic cells that CdTe-GSH QDs display, particularly when compared to Cys-CdTe [38], and the enhanced antibacterial effect of QDs and polymyxin B, CdTe-GSH QDs constitute potential candidates to improve the effect of clinical antimicrobials.

## Conclusion

**Figure 5:**
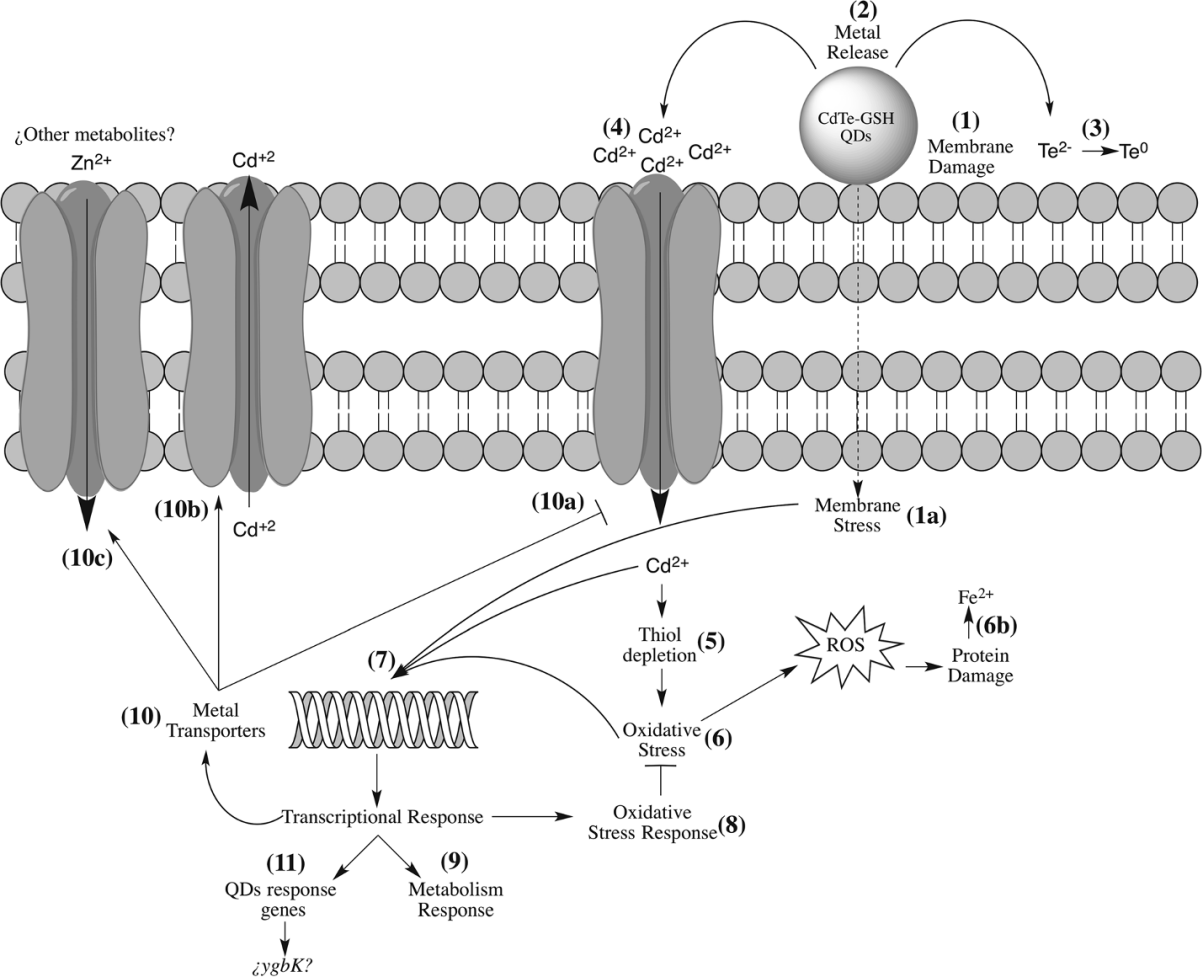
**Proposed mechanism of biomimetic CdTe-GSH QDs toxicity in** ***E. coli*** **.** Upon QDs exposure membrane damage is produced (1) with the concomitant generation of an envelope stress (1a). Furthermore, QDs are able to release metal/metalloid ions from the nanoparticle core, such as Cd^2+^ and Te^2^-(2). Since telluride (Te^2^-) present in CdTe QDs is easily oxidized into insoluble Te^0^, its contribution to QD toxicity is minor (3). Cadmium is released from the nanoparticle and enters the cell by divalent metal transporters (4). Once inside, metal affinity for reduced thiol groups produces RSH depletion (5). When the pool of oxidized thiols increase, the cell undergoes oxidative stress (6), which will increase ROS generation, resulting in protein damage and Fe^2+^ release (6b). All these processes generate a major transcriptional response associated with cadmium (7) and its secondary effects related with oxidative stress (8) and metabolic reconfiguration (9). Moreover, bacteria modulates the influx and efflux of toxic metals (10 a,b), promoting the exit of cadmium from the cell (*zntA*) and the entry of antioxidant molecules like Zn^2+^ (*znuA*) (10c). On the other hand, CdTe-GSH seem to elicit a QDs response mediated by *ybgK*, *clpS*, *hylC*, *yfcF*, *nrfC*, among others, which involves genes different from those modulated in response to cadmium stress (11). The *ybgK* gene is strongly up-regulated after both QDs treatment and has no known function.

Based on the transcriptomic, biochemical, microbiological and genetic results from this work, a general model of CdTe-GSH QDs toxicity in *E. coli* is proposed (Figure 5). When nanoparticles come into close contact with the bacterium an interaction with the cell envelope is established, and QDs generate a membrane stress that result in the modulation of several membrane transporters (eg. *ompF, ompW, malF, malM* and *malE*).

On the other hand, green and red QDs release metal ions (Cd^2+^ and Te^2-^) from the nanoparticle core when interacting with the bacterial cells. Tellurium does not significantly affect cells since it is present in NPs as telluride (Te^2-^), which in aqueous solution is rapidly oxidized to Te^0^, a more stable, insoluble, and much less toxic form of tellurium [68]. In contrast, cadmium represents an important source of toxicity, since it is released from the nanoparticle as Cd^2+^ upon cell interaction and can easily enter by a number of divalent metal transporters [69]. Once inside, cadmium affinity for reduced thiol groups results in thiol depletion and the cell undergoes oxidative stress [41]. Intracellular ROS increase can cause several forms of damage, like protein oxidation and the release of Fe^2+^ from iron-sulfur clusters [55].

All these effects generate a transcriptional response in *E. coli* that is mainly related to cadmium poisoning, oxidative stress and membrane damage. Bacteria promote Cd exit through ZntA and the entry of antioxidant molecules like Zn^2+^ (via ZnuA). Moreover, CdTe-GSH QDs seem to elicit a specific response mediated by these QDs (e.g. *ybgK, clpS*, *hylC*, *yfcF* and *nrfC*, among others), which involves genes that are not modulated during cadmium or oxidative stress. Among these, *ybgK* is one of the most interesting since it is strongly up-regulated after both green and red QDs treatment and has no known function to date.

Finally, given the interest in developing therapies and diagnostic tools based on QDs, these results are relevant to understanding the interaction of this kind of nanoparticles with bacterial cells. The fact that QDs-treated cells become more sensitive to polymyxin B could lead to the use of CdTe-GSH QDs as adjuvants in antimicrobial therapies. Green nanoparticles could be used in the diagnosis and treatment of bacterial pathogens susceptible to polymyxin B or other antibiotics acting similarly, as they do not display much toxicity while enhancing polymyxin B antibacterial activity.

## Methods

### Bacterial strains and growth media

*E. coli* BW25113 and strains from the KEIO mutant collection [70] were used in all experiments (Additional file 5: Table S1). Cells were grown in LB medium at 37°C with constant agitation using an overnight culture as pre-inoculum (1:100 dilution). Cells from the KEIO collection were grown in LB media supplemented with 30 μg/mL kanamycin.

### Synthesis of CdTe-GSH QDs

Green and red QDs were synthesized according to the protocol described by Pérez-Donoso et al*.* [39]. Briefly, a solution made of cadmium chloride (4 mM CdCl_2_), potassium tellurite (1 mM K_2_TeO_3_) and glutathione (15 mM GSH) in 15 mM borax-citrate buffer pH 9.4 was prepared. Afterwards, this solution was incubated in a water bath at 90°C and green and red QDs were obtained after 4 and 10 h incubation, respectively, as the reaction can be stopped at any time simply by incubating on ice or at 4°C. QDs solutions were dialyzed for 2 h against borax-citrate buffer pH 9.4 in order to eliminate non bound metal species. Afterwards, CdTe-GSH NPs were precipitated with two volumes of ethanol and centrifuged for 20 min at 13,000 × *g*. The resulting QDs were dried and weighted to obtain 100 mg/ml QDs solutions in borax citrate buffer pH 9.4. CdTe-GSH QDs in aqueous solution prepared by this method are stable and highly fluorescent for months at room temperature, 4°C or as powder after alcohol precipitation.

### DNA microarray experiments

Exponential *E. coli* cultures (OD_600_ ~ 0.5) were exposed for 15 min to 50 μg/mL red or green QDs and RNA was extracted using the RNeasy Mini kit (Qiagen), following the manufacturer’s instructions. The RNA was eluted and subjected to a second round of DNase I (Ambion Turbo DNA-free kit) treatment at 37°C for 30 min. RNA concentration and purity was determined using a Nanodrop 2000c spectrophotometer (Thermo).

Labeled cDNA probes were generated by reverse transcription using 20 μg of total RNA, SuperScript II (Invitrogen) and Alexa 555 and 647 dyes (Invitrogen). DNA microarrays slides were purchased from Microarrays Inc. and scanned in a ScanArray GX (Perkin Elmer) as described earlier [71]. GenePix Pro v6.0 software was used for image analysis. Limma package implemented in Bioconductor [72] was used to discount the background signal by the normexp method [73] and values were normalized using the LOESS procedure [74]. T-test was used to identify those genes whose change in expression was significant and 3 criteria (M value, A value and p value from t-test) were used for determining differential expression. The threshold for genes to be considered were values of M ≥ 2 (induction), M ≤ -2 (repression); A ≥ 8 and p ≤ 0.05. All genes that showed differential expression were categorized by Gene Ontology associations (The Gene Ontology Consortium, 2000) using biological process term. By using a custom python script with all Gene Ontology terms, a GO plot based on Ecocyc webpage was constructed and classified [75].

### Real time quantitative RT-PCR

qRT-PCR was performed using the primers listed in Additional file 6: Table S2 as previously described [67], with a minor modification of the PCR program. Briefly, relative quantification was performed using a Brilliant II SYBR Green QPCR Master Reagent Kit and the Mx3000P detection system (Stratagene). 16S rRNA was used for normalization. The reaction mixture was carried out in a final volume of 20 μl containing 1 μl of diluted cDNA (1:1000), 0.24 μl of each primer (120 nM), 10 μl of 2 x Master Mix, 0.14 μl of diluted ROX (1:200) and 8.38 μl of H_2_O. The reaction was performed under the following conditions: 10 min at 95°C followed by 40 cycles of 30 s at 95°C, 30 s at 58°C and 30 s at 72°C. Finally, a melting cycle from 65°C to 95°C was performed to check for amplification specificity. Amplification efficiency was calculated from a standard curve constructed by amplifying serial dilutions of RT-PCR products for each gene. These values were used to obtain the fold-change in expression for the gene of interest normalized with 16S levels according to Pfaffl [76].

### Minimal inhibitory concentrations (MICs)

MIC determinations were performed in 96 well microplates prepared aseptically adding LB medium and QDs at the desired concentration by serial dilution in a final volume of 150 μL. *E. coli* cells were grown to OD_600_ ~ 0.5 and then diluted 10-fold. Then, 10 μL of the diluted cell suspension were added to each well and the plate was incubated at 37°C for 24 h. MIC was determined as the concentration where the OD_600_ was less than or equal to 50% of the absorbance obtained in the untreated control. Each assay was performed in triplicate.

### Flow cytometry assays

Exponential *E. coli* cultures (OD_600_ ~ 0.5) were exposed for 30 min to 50 μg/mL red or green QDs or 10 mM H_2_O_2_. Samples were washed with PBS 1X buffer twice and then were incubated with 2’, 7’-dichlorofluorescein diacetate (H_2_DCFDA, for ROS detection) or propidium iodide (PI, to measure membrane damage) for 10 min. The fluorescence-activated cell sorting (FACS) data was recorded with a BD Biosciences Accuri C6 flow cytometer. H_2_DCFDA and PI fluorescence were excited with a 488 nm argon laser. Emissions were detected with FL1-A (using FL1 emission filter 533/30) and FL3-A (using FL3 emission filter 610/20). Flow cytometry data was analyzed using Kaluza Analysis 1.3.

### Metal quantification on QDs-treated cells

Metal quantification experiments were performed as previously described by Montes et al., [77], with some modifications: *E. coli* was grown at 37°C to OD_600_ ~ 0.5 and cultures were amended with 50 μg/mL freshly-synthesized green or red CdTe-GSH QDs. After incubating for 15, 60 or 120 min, cells were sedimented at 10 000 *x g* for 6 min. Supernatants were discarded, pellets were suspended in 1 mL of 1 N HNO_3_ and allowed to dissolve overnight at room temperature. Samples were diluted 1:10 with 1 N HNO_3_ and centrifuged at 10 000 *x g* for 6 min. Supernatants were used for cadmium and tellurium quantification by inductively coupled plasma atomic emission spectrometry, ICP-AES (Spectro CIROS Vision ICP-OES) using 1 N HNO_3_ as matrix. Calibration curves were constructed using cadmium and tellurium commercially available ICP standards.

### *in vitro*quantification of Cd released from QDs

Red and green QDs were diluted to 1 000 μg/mL solutions with sterile distilled water. To evaluate cadmium release, QDs solutions were incubated at room temperature for 10 min, mixed with isopropanol (1:1) and centrifuged at 12 000 *x g* for 10 min to separate the nanoparticles from the soluble cadmium fraction. Supernatants were diluted 1:10 with sterile distilled water and used for metal quantification by flame absorption atomic spectrometry (FAAS) using an AA-260 flame atomic absorption spectrometer (Shimadzu).

### Viability assay

Viability assays were performed on *E. coli* wild type and KEIO mutant strains grown to OD_600_ ~ 0.5 and exposed to 50 μg/mL of red or green QDs for 20 min. After treatment, serial dilutions of all strains analyzed were plated on LB agar and colony forming units (CFU) were determined after 24 h.

### Antibiotic susceptibility assays

*E. coli* was grown in LB medium at 37°C with constant agitation to OD_600_ ~ 0.5. Then, red or green QDs were added to the cultures at a final concentration of 50 μg/mL and incubated for 15 min. In parallel, a 15 min pre-treatment with 5 and 50 μg/mL CdCl_2_ was performed. After pre-treatments, cells were washed twice with LB medium and used for tetracycline and polymyxin B MIC determination, as described above.

### Statistical analysis

All experiments were performed in three biological and technical replicates. The statistical analyses used the one-way or two-way ANOVA with a post-hoc Bonferroni’s test. Differences were considered significant at p values of ≤ 0.05 for all statistical analyses.

## Availability of supporting data

The DNA microarray data discussed in this study have been deposited in NCBI Gene Expression Omnibus (GEO; http://www.ncbi.nlm.nih.gov/geo/), and are accessible through GEO series accession no. GSE58912.

http://www.ncbi.nlm.nih.gov/geo/query/acc.cgi?token=qjivcmoyzhqnzcr&acc=GSE58912.

## Electronic supplementary material

Additional file 1: TableS3: Genes regulated in response to red QDs. (DOCX 92 KB)

Additional file 2: Table S4: Genes regulated in response to green QDs. (DOCX 90 KB)

Additional file 3: Figure S1: Validation of microarray data using qRT-PCR of randomly selected genes. Total RNA was extracted from wild type strain grown aerobically in LB media until OD_600_ ~ 0.5 and treated with red QDs (A) or green QDs (B) for 15 min to analyze the expression by qRT-PCR. Values are based on fold change (Control/QDs treated) calculated from ∆∆Ct values and log_2_ transformed. All genes present statistically significant differences between control (untreated) and QDs (red or green) treated cells (p < 0.05). Data represent the means ± standard deviations (n = 3). (TIFF 129 KB)

Additional file 4: Table S5: Genes regulated by both red and green QDs. (DOCX 82 KB)

Additional file 5: Table S1: Bacterial strains used in this study. (DOCX 42 KB)

Additional file 6: Table S2: Primers used in this study. (DOCX 109 KB)

## Abbreviations

QDs: Quantum Dots
CdTe: Cadmium telluride
GSH: Glutathione
MIC: Minimal inhibitory concentration
ICP-AES: Inductively coupled plasma atomic emission spectrometry
ROS: Reactive oxygen species
LB: Luria Bertani.
