# Supplementary material for: Microarray analysis of the Escherichia coli response to CdTe-GSH Quantum Dots: understanding the bacterial toxicity of semiconductor nanoparticles
Source: BMC Genomics. 2014 Dec 12;15(1):1099. doi: 10.1186/1471-2164-15-1099 (PMC4300170; doi:10.1186/1471-2164-15-1099)
Supplement: Supplementary file 1 — Additional file 1: TableS3: Genes regulated in response to red QDs. (DOCX 92 KB) [file 12864_2014_6802_MOESM1_ESM.docx]

**Supplementary Table 3.** Genes regulated in response to red QDs.

|  | **Up-regulated** |  |
| --- | --- | --- |
| **Gene** | **Description** | **Fold Change** |
| *soxS* | DNA-binding transcriptional dual regulator | 8,063 |
| *ybgK* | predicted enzyme subunit | 5,547 |
| *yeaN* | predicted transporter | 4,014 |
| *znuA* | high-affinity zinc transporter periplasmic component | 3,564 |
| *fruB* | fused fructose-specific PTS enzymes: IIA | 3,472 |
| *miaA* | tRNA delta(2)-isopentenylpyrophosphate transferase | 3,204 |
| *zntA* | zinc, cobalt and lead efflux system | 3,058 |
| *lysU* | lysine tRNA synthetase, inducible | 3,038 |
| *kpsT* | kpsT protein | 2,845 |
| *adhE* | fused acetaldehyde-CoA | 2,744 |
| *dnaK* | molecular chaperone DnaK | 2,682 |
| *ftn* | ferritin iron storage protein (cytoplasmic) | 2,645 |
| *ygfX* | hypothetical protein | 2,537 |
| *kdpE* | DNA-binding response regulator in two-component regulatory system with KdpD | 2,471 |
| *nfrA* | bacteriophage N4 receptor, outer membrane subunit | 2,453 |
| *ycfQ* | hypothetical protein | 2,448 |
| *hfq* | RNA-binding protein Hfq | 2,386 |
| *yafK* | hypothetical protein | 2,322 |
| *yhbW* | predicted enzyme | 2,249 |
| *ycgF* | predicted FAD-binding phosphodiesterase | 2,237 |
| *cdaR* | DNA-binding transcriptional activator | 2,201 |
| *ppiA* | peptidyl-prolyl cis-trans isomerase A (rotamase A) | 2,193 |
| *ydfP* | hypothetical protein ydfP precursor | 2,193 |
| *sdaA* | L-serine deaminase I | 2,193 |
| *minD* | membrane ATPase of the MinC-MinD-MinE system | 2,161 |
| *yfdZ* | hypothetical protein | 2,156 |
| *clpB* | protein disaggregation chaperone | 2,142 |
| *tus* | DNA replication terminus site-binding protein | 2,137 |
| *trxC* | thioredoxin 2 | 2,084 |
| *clpS* | ATP-dependent Clp protease adaptor protein ClpS | 2,076 |
| *mtlD* | mannitol-1-phosphate 5-dehydrogenase | 2,075 |
| *frmR* | regulator protein that represses frmRAB operon | 2,074 |
| *yecD* | predicted hydrolase | 2,073 |
| *marR* | DNA-binding transcriptional repressor of multiple antibiotic resistance | 2,070 |
| *fabG* | 3-ketoacyl-(acyl-carrier-protein) reductase | 2,056 |
| *yeaC* | hypothetical protein | 2,042 |
| *ygaV* | predicted DNA-binding transcriptional regulator | 2,031 |
| *ydjL* | predicted oxidoreductase, Zn-dependent and NAD(P)-binding | 2,030 |
| *yihF* | hypothetical protein | 2,016 |
| *yfcF* | predicted enzyme | 2,015 |
| *wzx* | O antigen flippase Wzx | 2,005 |
| *glnA* | glutamine synthetase | 2,002 |
| *pinR* | Rac prophage; predicted site-specific recombinase | 2,002 |
|  |  |  |
|  | **Down-regulated** |  |
| **Gene** | **Description** | **Fold Change** |
| *gmr* | modulator of Rnase II stability | -2,002 |
| *nrfC* | formate-dependent nitrite reductase, 4Fe4S subunit | -2,003 |
| *lldP* | L-lactate permease | -2,015 |
| *mraY* | phospho-N-acetylmuramoyl-pentapeptide- transferase | -2,020 |
| *ureG* | putative urease accessory protein G \| putative urease accessory protein G | -2,021 |
| *ilvN* | acetolactate synthase small subunit | -2,023 |
| *ydcF* | hypothetical protein | -2,035 |
| *lamB* | maltoporin precursor | -2,040 |
| *yeiH* | conserved inner membrane protein | -2,048 |
| *hlyD* | hemolysin D | -2,057 |
| *yidQ* | hypothetical protein | -2,058 |
| *cspI* | Qin prophage; cold shock protein | -2,059 |
| *sucA* | alpha-ketoglutarate decarboxylase | -2,060 |
| *xylR* | DNA-binding transcriptional activator, xylose-binding | -2,060 |
| *ampC* | beta-lactamase/D-alanine carboxypeptidase | -2,075 |
| *paaD* | predicted multicomponent oxygenase/reductase subunit for phenylacetic acid degradation | -2,091 |
| *hisQ* | histidine/lysine/arginine/ornithine transporter subunit | -2,116 |
| *lldR* | DNA-binding transcriptional repressor | -2,122 |
| *yehR* | hypothetical lipoprotein yehR precursor | -2,139 |
| *yfaA* | hypothetical protein | -2,140 |
| *yjfM* | hypothetical protein | -2,153 |
| *yjbA* | phosphate-starvation-inducible protein PsiE | -2,180 |
| *yahL* | hypothetical protein | -2,182 |
| *glpK* | glycerol kinase | -2,211 |
| *sgbU* | predicted L-xylulose 5-phosphate 3-epimerase | -2,223 |
| *malP* | maltodextrin phosphorylase | -2,230 |
| *malF* | maltose transporter subunit | -2,233 |
| *asnC* | DNA-binding transcriptional dual regulator | -2,247 |
| *cysW* | sulfate/thiosulfate transporter subunit | -2,264 |
| *glpF* | glycerol facilitator | -2,346 |
| *glpT* | sn-glycerol-3-phosphate transporter | -2,359 |
| *ydaU* | Rac prophage; conserved protein | -2,369 |
| *dgoD* | galactonate dehydratase | -2,404 |
| *gltI* | glutamate and aspartate transporter subunit | -2,458 |
| *yhhA* | hypothetical protein | -2,485 |
| *cesD* | cesD | -2,544 |
| *malE* | maltose ABC transporter periplasmic protein | -2,565 |
| *malM* | maltose regulon periplasmic protein | -2,596 |
| *thiC* | thiamine biosynthesis protein ThiC | -2,623 |
| *yghX* | predicted hydrolase (pseudogene) | -2,696 |
| *yqiJ* | predicted inner membrane protein | -2,707 |
| *yrdB* | hypothetical protein | -3,006 |
| *ompF* | outer membrane porin 1a (Ia;b;F) | -3,111 |
| *chbC* | N,N'-diacetylchitobiose-specific enzyme IIC component of PTS | -3,167 |
| *wza* | lipoprotein required for capsular polysaccharide translocation through the outer membrane | -3,262 |
| *ykfB* | CP4-6 prophage; predicted protein | -3,283 |
| *nikA* | nickel transporter subunit | -3,467 |
| *cspA* | major cold shock protein | -4,006 |
| *cspG* | DNA-binding transcriptional regulator | -4,440 |
| *dgoR* | predicted DNA-binding transcriptional regulator | -4,717 |
| *cspB* | Qin prophage; cold shock protein | -5,658 |
| *nikD* | ATP-binding protein of nickel transport system | -6,544 |
