# Supplementary material for: Microarray analysis of the Escherichia coli response to CdTe-GSH Quantum Dots: understanding the bacterial toxicity of semiconductor nanoparticles
Source: BMC Genomics. 2014 Dec 12;15(1):1099. doi: 10.1186/1471-2164-15-1099 (PMC4300170; doi:10.1186/1471-2164-15-1099)
Supplement: Supplementary file 2 — Additional file 2: Table S4: Genes regulated in response to green QDs. (DOCX 90 KB) [file 12864_2014_6802_MOESM2_ESM.docx]

**Supplementary Table 4.** Genes regulated in response to green QDs.

|  | **Up-regulaed** |  |
| --- | --- | --- |
| **Gene** | **Description** | **FoldChange** |
| *ybgK* | predicted enzyme subunit | 5,458 |
| *znuA* | high-affinity zinc transporter periplasmic component | 4,163 |
| *ycgF* | hypothetical protein ycgF | 3,421 |
| *yecE* | hypothetical protein | 3,059 |
| *tus* | DNA replication terminus site-binding protein | 2,498 |
| *hycI* | enzyme; degradation of proteins, peptides, glyco | 2,497 |
| *gsk* | inosine/guanosine kinase | 2,389 |
| *pyrL* | pyrBI operon leader peptide | 2,301 |
| *idnD* | L-idonate 5-dehydrogenase, NAD-binding | 2,267 |
| *ynaA* | Rac prophage; predicted tail protein | 2,240 |
| *manC* | mannose-1-P guanosyltransferase | 2,237 |
| *zntA* | zinc, cobalt and lead efflux system | 2,229 |
| *yhcH* | hypothetical protein | 2,175 |
| *ybhL* | predicted inner membrane protein | 2,117 |
| *ydgI* | predicted arginine/ornithine antiporter transporter | 2,042 |
| *nrfB* | nitrite reductase, formate-dependent, penta-heme cytochrome c | 2,032 |
|  |  |  |
|  | **Down-regulated** |  |
| **Gene** | **Description** | **Fold Change** |
| *htrB* | lipid A biosynthesis lauroyl acyltransferase | -2,004 |
| *lysR* | DNA-binding transcriptional dual regulator | -2,029 |
| *sfaB* | putative F1C and S fimbrial switch regulatory protein | -2,039 |
| *gltI* | glutamate and aspartate transporter subunit | -2,043 |
| *ydfZ* | hypothetical protein | -2,059 |
| *ompW* | outer membrane protein W | -2,076 |
| *yieK* | hypothetical protein | -2,093 |
| *yohK* | predicted inner membrane protein | -2,113 |
| *racC* | Rac prophage; predicted protein | -2,124 |
| *ykiA* | hypothetical protein | -2,151 |
| *stfR* | Rac prophage; predicted tail fiber protein | -2,160 |
| *lacI* | lac repressor | -2,174 |
| *yiaO* | putative ABC transporter periplasmic binding protein yiaO precursor | -2,178 |
| *mchF* | probable microcin H47 secretion ATP-binding protein | -2,230 |
| *ilvG* | acetolactate synthase II large subunit | -2,232 |
| *aroD* | 3-dehydroquinate dehydratase | -2,233 |
| *terZ* | putative phage inhibition, colicin resistance and tellurite resistance protein | -2,236 |
| *ygeM* | hypothetical protein | -2,238 |
| *yecT* | hypothetical protein | -2,388 |
| *eutG* | predicted alcohol dehydrogenase in ethanolamine utilization | -2,453 |
| *yffQ* | CPZ-55 prophage; predicted protein | -2,690 |
| *yhaJ* | predicted DNA-binding transcriptional regulator | -3,035 |
| *feoB* | ferrous iron transport protein B | -3,372 |
| *nikD* | ATP-binding protein of nickel transport system | -4,127 |
| *lolB* | outer membrane lipoprotein LolB precursor | -4,505 |
| *guaD* | guanine deaminase | -5,540 |
