# Supplementary figures and images for: Microarray analysis of the Escherichia coli response to CdTe-GSH Quantum Dots: understanding the bacterial toxicity of semiconductor nanoparticles
Source: BMC Genomics. 2014 Dec 12;15(1):1099. doi: 10.1186/1471-2164-15-1099 (PMC4300170; doi:10.1186/1471-2164-15-1099)

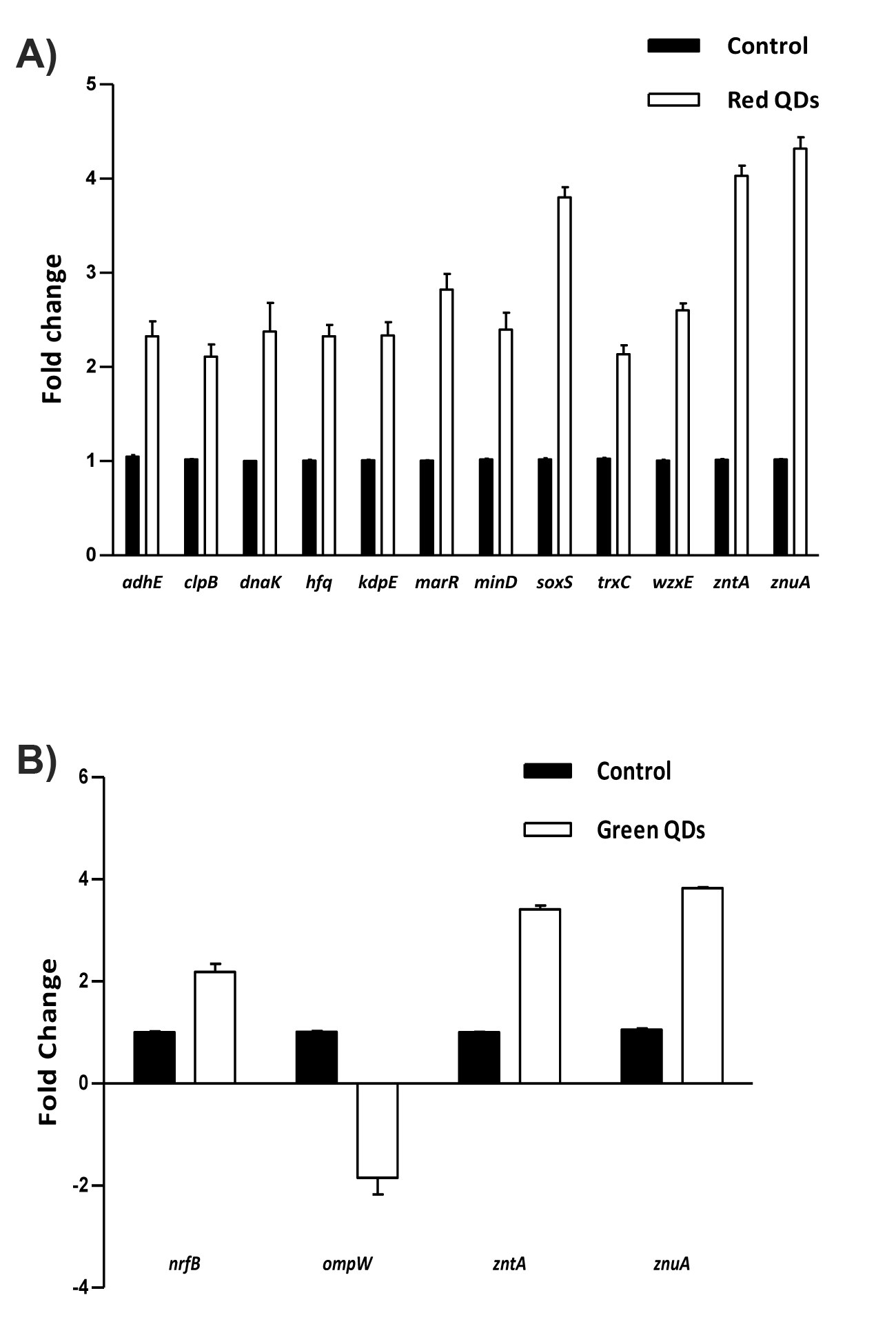

Supplement: Supplementary file 3 — Additional file 3: Figure S1: Validation of microarray data using qRT-PCR of randomly selected genes. Total RNA was extracted from wild type strain grown aerobically in LB media until OD600 ~ 0.5 and treated with red QDs (A) or green QDs (B) for 15 min to analyze the expression by qRT-PCR. Values are based on fold change (Control/QDs treated) calculated from ∆∆Ct values and log2 transformed. All genes present statistically significant differences between control (untreated) and QDs (red or green) treated cells (p < 0.05). Data represent the means ± standard deviations (n = 3). (TIFF 129 KB) [file 12864_2014_6802_MOESM3_ESM.tiff]
