# Supplementary material for: Microarray analysis of the Escherichia coli response to CdTe-GSH Quantum Dots: understanding the bacterial toxicity of semiconductor nanoparticles
Source: BMC Genomics. 2014 Dec 12;15(1):1099. doi: 10.1186/1471-2164-15-1099 (PMC4300170; doi:10.1186/1471-2164-15-1099)
Supplement: Supplementary file 4 — Additional file 4: Table S5: Genes regulated by both red and green QDs. (DOCX 82 KB) [file 12864_2014_6802_MOESM4_ESM.docx]

**Supplementary Table 5.** Genes regulated by both red and green QDs**.**

| **Genes regulated in response to both QDs** | | |
| --- | --- | --- |
| **Gene** | **Description** | **Go annotation** |
| *ybgK* | predicted enzyme subunit | 0016829 |
| *znuA* | high-affinity zinc transporter periplasmic component | 0007155 |
| *zntA* | zinc, cobalt and lead efflux system | 0005886/0006200/0006824/0006829/0008551/0010312/0015087/0015094/0015099/0015692/0016021/0016463/0016887/0019829/0030001/0035444/0046686/0046872/0070574/0071577 |
| *ycgF* | predicted FAD-binding phosphodiesterase | 0009637/0009882/0018298/0043433/0050660/0070491/0071949 |
| *tus* | DNA replication terminus site-binding protein | 0003677/0005737/0006274/0071807 |
| *gltI* | glutamate and aspartate transporter subunit | 0005215/0006865/0042597 |
| *nikD* | nickel transporter subunit | 0005524/0015413/0016151/0043190 |
