# Supplementary material for: Microarray analysis of the Escherichia coli response to CdTe-GSH Quantum Dots: understanding the bacterial toxicity of semiconductor nanoparticles
Source: BMC Genomics. 2014 Dec 12;15(1):1099. doi: 10.1186/1471-2164-15-1099 (PMC4300170; doi:10.1186/1471-2164-15-1099)
Supplement: Supplementary file 5 — Additional file 5: Table S1: Bacterial strains used in this study. (DOCX 42 KB) [file 12864_2014_6802_MOESM5_ESM.docx]

**Supplementary Table 1.** Bacterial strains used in this study.

| **Strain** | **Relevant characteristic(s)** | **Source or reference** |
| --- | --- | --- |
| *E. coli* BW25113 | Wild type | Laboratory |
| Δ*zntA* | *zntA* mutant of KEIO collection | Baba *et al*., 2006 |
| Δ*trxC* | *trxC* mutant of KEIO collection | Baba *et al*., 2006 |
| Δ*soxS* | *soxS* mutant of KEIO collection | Baba *et al*., 2006 |
