# Supplementary material for: Microarray analysis of the Escherichia coli response to CdTe-GSH Quantum Dots: understanding the bacterial toxicity of semiconductor nanoparticles
Source: BMC Genomics. 2014 Dec 12;15(1):1099. doi: 10.1186/1471-2164-15-1099 (PMC4300170; doi:10.1186/1471-2164-15-1099)
Supplement: Supplementary file 6 — Additional file 6: Table S2: Primers used in this study. (DOCX 109 KB) [file 12864_2014_6802_MOESM6_ESM.docx]

**Supplementary Table 2.** Primers used in this study.

| Primer Name | Sequence |
| --- | --- |
| 16S_Fw | 5’ GGCCGCAAGGTTAAAACTCAAATG 3’ |
| 16S_Rv | 5’ AACCGCTGGCAACAAAGGATAAGG 3’ |
| adhE_Fw | 5’ GCTGAACTTAACGCACTCGTAG 3’ |
| adhE_Rv | 5’ GTTCAGCGATAGTGATGGTACC 3’ |
| clpB_Fw | 5’ TTCCAGCTTGCTCTTGCCGA 3’ |
| clpB_Rv | 5’ AAGTGCCGCCAGAACGAACA 3’ |
| dnaK_Fw | 5’ ATTATGGATGGCACCACTCCT 3’ |
| dnaK_Rv | 5’ CAGCACTTCAGCAGAAATCTG 3’ |
| feoB_Fw | 5’ TACATTTTGAGTGGCGACGC 3’ |
| feoB_Rv | 5’ GTCGGAAGGCATCACTTTTG 3’ |
| ftn_Fw | 5’ TGGAACTGTACTCTTCACTGC 3’ |
| ftn_Rv | 5’ TACAGACCTTCGCCGCTTTT 3’ |
| hfq_Fw | 5’ TGGTATTAAGCTGCAAGGGC 3’ |
| hfq_Rv | 5’ GTTGCGCGGAAGTATTCTGC 3’ |
| kpdE_Fw | 5’ TATTCGTCGCTTTCTGCGCA 3’ |
| kpdE_Rv | 5’ AATCACGCGGGCGGCTAAAT 3’ |
| marR_Fw | 5’ GGGTCGCTTAATCCATATGGT 3’ |
| marR_Rv | 5’ AGGTCCTGGCCAACTAATTG 3’ |
| minD_Fw | 5’ TTGGTAAGACAACCTCCAGC 3’ |
| minD_Rv | 5’ ATAGAGTGCCATTAACGCACC 3’ |
| nfrB_Fw | 5’ TTATTAACTGCCGGGGTGCT 3’ |
| nfrB_Rv | 5’ AGGACATACAGACGCTGTTC 3’ |
| ompF_Fw | 5’ TTCTGGCAGTGATCGTCCCT 3’ |
| ompF_Rv | 5’ GCCGTAATCGAAAGAACCAAC 3’ |
| ompW_Fw | 5’ GGCAGTAACAACTCTTCTCTC 3’ |
| ompW_Rv | 5’ CAACGTAAGGACGGAATTTGC 3’ |
| soxS_Fw | 5’ GGATCTTATCGCATGGATTGAC 3’ |
| soxS_Rv | 5’ AGGCGGTGGCGATAATCGCT 3’ |
| trxC_Fw | 5’ TACCCATTGTCAGGCCATCAA 3’ |
| trxC_Rv | 5’GCTCAATTCACGTTCAGCTTC 3’ |
| wzxE_Fw | 5’ TCAAGATTGGTGCCGGGTTA 3’ |
| wzxE_Rv | 5’ GCCCCTGATAGTCGGTATTA 3’ |
| ydjL_Fw | 5’ TTGATGTCCCACAACCCATG 3’ |
| ydjL_Rv | 5’ GAGAATTTCACCAGGAACCAG 3’ |
| zntA_Fw | 5’ TCCTGACAATCACGGCAAGA 3’ |
| zntA_Rv | 5’ CGCACGGCATTTTCTACCTT 3’ |
| znuA_Fw | 5’ TTTCGCAGCATTATCCGCCG 3’ |
| znua_Rv | 5’ GCGGTTTCACATCTTCAAGCT 3’ |
